# Supplementary material for: NKCC1 and KCC2 Chloride Transporters Have Different Membrane Dynamics on the Surface of Hippocampal Neurons
Source: Cells. 2023 Sep 26;12(19):2363. doi: 10.3390/cells12192363 (PMC10571912; doi:10.3390/cells12192363)
Supplement: Supplementary file 1 [file cells-12-02363-s001.zip › cells-2596349-supplementary.pdf]

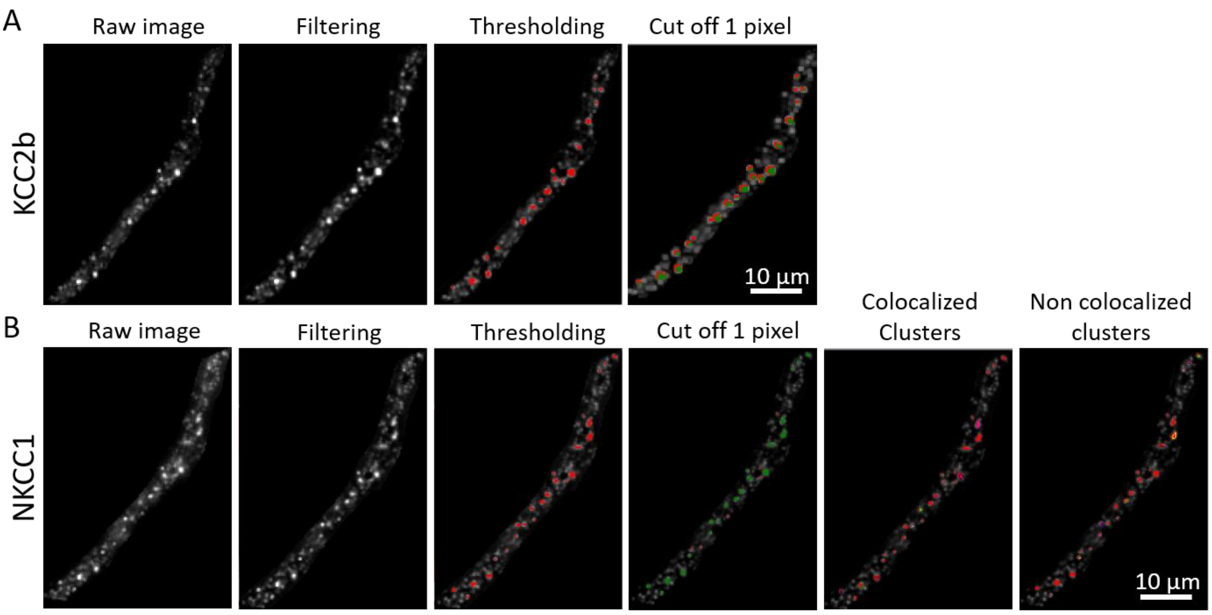

Figure S1. Method of analysis of NKCC1 clustering and NKCC1/KCC2 colocalization using homemade routine on Metamorph. Quantification is shown here on a representative neuron transfected with KCC2-Flag and NKCC1b-HA at 14 DIV and stained for Flag and HA at 21 DIV.

A, A region of interest (ROI) drawn along the primary dendrite of a neuron is extracted from a raw KCC2-Flag image and then flattened and background filtered (kernel size, 3 X 3 X 2) (Filtering) to enhance cluster outlines, and a user defined intensity threshold is applied to select KCC2 clusters (red in Thresholding image) and avoid their coalescence. Only KCC2 clusters ≥ 1 pixel (green in Cut off 1 pixel image) are taken into account for further analysis. Scale bar, 10 µm.

B, The ROI is then loaded on the NKCC1b image; the dendritic region is extracted and processed for filtering, thresholding and cluster selection as in A. Then, for quantification of NKCC1b and KCC2 colocalization, NKCC1b clusters colocalized on at least 1 pixel with KCC2 clusters are considered for colocalization. Clusters colocalized and non colocalized clusters were then outlined and the corresponding

regions were transferred onto raw images to determine the mean cluster number, area and fluorescence intensity. Scale bar, 10 µm.

For this particular example, the program detected 20 NKCC1b and 18 KCC2 clusters. 50% of NKCC1b clusters were found colocalized with KCC2 clusters. All NKCC1b and KCC2 clusters occupy 3.14% and 4.08% of the dendritic surface and NKCC1/KCC2 colocalized clusters occupy 1.83% of the membrane.

Related to Materials and Methods and Figure 3.
